# Accuracy of Telehealth Visits for Acute Care Needs in Family Medicine

**DOI:** 10.7759/cureus.59569

**Published:** 2024-05-03

**Authors:** Bryan A Farford, Ellen M Bulbarelli, Inyam Ricketts, Sahil Nath, Abhimanyu S Ahuja, Josh Keith

**Affiliations:** 1 Family Medicine, Mayo Clinic, Jacksonville, USA; 2 Medicine, Florida Atlantic University Charles E. Schmidt College of Medicine, Boca Raton, USA

**Keywords:** accurate diagnosis, virtual provider visit, outpatient family medicine, acute care model, telehealth appointments

## Abstract

Introduction

As primary care practices transition to a post-pandemic system of healthcare, it is important to recognize the benefits of offering telehealth services. Little research is available on the effectiveness of telehealth visits for managing acute illnesses or conditions in primary care practice.

Methods

Using the reporting functionality in the Epic™ electronic health record (EHR) (Epic Systems Corporation, Verona), a report was generated to identify all telehealth visit encounters that were completed in a family medicine clinic from March 1, 2020, to June 30, 2020. The report identified patients who had an acute complaint and required an in-office visit within 60 days of the telehealth encounter. If the patient required a face-to-face visit, that was not directed by the provider, the chart was reviewed to determine whether the diagnosis changed. The primary outcome was returning for a face-to-face visit within 30 days of the telehealth visit for the same acute need.

Results

The cohort included 349 telehealth visits for 303 patients. For patients who had more than one telehealth visit, only the first one was included in the analysis. Among the 303 patients, 50 (16.5%) returned for a face-to-face visit within 30 days of the telehealth visit (95% confidence interval: 12.5%-21.2%), and 71 (23.6%) returned for a telehealth visit within 60 days (95% confidence interval: 18.9%-28.8%). Furthermore, 19 of the 50 patients (38%) that returned for a face-to-face visit did not have a change in diagnosis, and, in some instances, the diagnosis made on the telehealth visit was only slightly different from the face-to-face visit.

Discussion and conclusion

Telehealth, specifically two-way, synchronous, interactive patient-provider communication through audio and video equipment, for acute care needs in a primary care practice helps reduce the need for in-person visits and can address patient complaints without the need for in-person follow-up.

## Introduction

The COVID-19 pandemic changed the way physicians and healthcare workers practice medicine. Healthcare systems had to adapt to overcome the remarkable challenges faced during this time, including a shortage of equipment and increased patient loads [1]. A significant number of patient needs went unmet, as many primary care clinics stopped seeing patients with upper respiratory symptoms to reduce the spread of infection. As a result, telehealth visits have increasingly been promoted as a viable method of providing medical care while allowing patients to receive their care outside of an office setting [1]. Increased telehealth visits also helped conserve medical supplies, manage the surge in demand for clinical services, and reduce community and nosocomial spread [2]. Beyond its value in a pandemic, telehealth has also been shown to reduce costs, save time for patients, improve patients’ quality of life, and provide access to care in remote areas [3]. While telehealth options existed before the pandemic, many health systems reported low rates of utilization [2]. However, between March and April 2020, telehealth use increased from less than 1% of visits to as much as 80% in certain areas of the US. Data from a 2021 national survey found that telehealth use was commonly utilized, with 23% of respondents reporting a telehealth visit in the four weeks before the survey [4].

As primary care practices transition to a post-pandemic system of healthcare, it is important to recognize the benefits of offering telehealth services. Three major obstacles to accessing primary care are physical disabilities, inability to take time off work to attend appointments, and geographic and transportation-related barriers [5]. The utilization of telehealth can provide a pathway to overcome these challenges by improving access to primary care needs. The development of a robust telehealth network can also help expand care to patients in previously underserved areas [2].

A telehealth system with a strong and sustainable infrastructure can allow for a more proficient use of resources and staff [2]. Telehealth does not require additional physical space to provide care. In traditional primary care settings, physicians are limited by the number of exam rooms and office space available within their clinic. Traditionally, to grow a primary care clinic, more physical space was required to add more healthcare staff and to see more patients. With telehealth, no additional exam rooms are needed, and many times physicians and rooming staff can work remotely, which frees up office space.

Previous studies have illustrated the benefits of telehealth in managing chronic conditions such as mental health issues, diabetes, and congestive heart failure (CHF) [6-8]. However, research is limited on the effectiveness of telehealth visits for managing acute illnesses or conditions in a primary care practice. One research study that assessed urgent care telehealth for students 18 years or younger found that most students returned to the classroom without the need for further follow-up [9]. With primary care providers being the first contact for nearly half of the United States’ acute care needs, it is important to understand the effectiveness of telehealth services to address such needs [10].

The main domains to consider when practicing telehealth are convenience, efficiency, communication, privacy, and comfort, especially in the primary care setting [11]. While some studies suggest that telemedicine interventions tend to be at least as effective as traditional care, data on overall outcomes are limited [12]. In the primary care setting, telemedicine may serve as a feasible option to manage acute complaints while diverting patients from in-person visits [1]. Despite the changes in healthcare and the benefits of telehealth, there are still questions on whether it can be completely effective and reduce the need for in-office, face-to-face visits for acute needs. Moreover, it is important to ensure that patients using telehealth for acute care complaints receive an accurate diagnosis and treatment plans to prevent adverse outcomes.

The primary aim of this descriptive, retrospective chart review study was to estimate the proportion of patients with a telehealth visit for an acute care need who required a face-to-face visit for the same acute care need within 30 days of the telehealth visit. Additionally, an assessment of whether a change in diagnosis occurred during the face-to-face visit.

A secondary aim of the study is to identify what acute needs are best suited for telehealth visits. For this study, telehealth visits are two-way, synchronous, interactive patient-provider communication through audio and video equipment.

## Materials and methods

Before initiating the study, Mayo Clinic Institutional Review Board (IRB) approval was obtained (ID: 20-005367).

Using the reporting functionality in the Epic™ electronic health record (EHR) (Epic Systems Corporation, Verona, USA), a report was generated to identify all telehealth visit encounters that were completed in a nonprofit family medicine department from March 1, 2020, to June 30, 2020. The department comprised four distinct clinics that were each in a metropolitan area and were physically separated from each other by an average of 20 miles. The department had 30,000 female patients, 23,000 male patients, and four that identified as nonbinary. Patients less than 18 comprised 5% of the department, patients between the ages of 18-65 comprised 70% of the department, and the remaining 25% were 65 years or older. Approximately 70% of the patients had nongovernment insurance. During this time, patients were encouraged to have a telehealth visit versus an in-office visit because of the COVID-19 pandemic. All patients were scheduled over the telephone by a nonclinical scheduling staff member.

Additionally, the report included the patient’s name, medical record number, date of encounter, and the diagnoses that were coded for that visit. Upon review of the data, encounters that did not include an acute diagnosis code were removed from the report. Further chart review was required in some instances when it was not clear whether the diagnosis was acute or chronic.

Once the initial report was complete, a second report was generated using a pivot function that identified those patients that had a subsequent visit within 30 and then 60 days of the initial telehealth visit encounter. This report included in-office visits, emergency department visits, and telehealth visits. The report also generated the date of the encounters and the diagnoses coded during the encounter.

Using this report, a manual chart review was performed to identify whether the subsequent visits were related to the initial telehealth visit encounter. The chart review also assessed whether the patient was directed to follow up in the office during the initial telehealth visit. If the patient required a face-to-face visit, that was not directed by the provider, the chart was reviewed to determine whether the diagnosis changed. An exploratory analysis of the data was done, and a search for specific keywords to was conducted identify the most common diagnoses.

Any patient that had a telehealth visit for a new acute need between March 1, 2020, and June 30, 2020, were included. Patients who had telehealth visits for chronic condition management or who were directed to have a follow-up visit via telehealth were excluded.

It was planned to have a sample size of at least 200 patients meeting the eligibility criteria during the study period. For the primary analysis, it was estimated the two-sided 95% confidence interval (CI) (Wilson method) for the proportion of patients receiving a video visit for an acute care need who required a face-to-face visit within 30 days of the video visit for the same acute care need.

The primary outcome was returning for a face-to-face visit within 30 days of the telehealth visit for the same acute need. All analyses were done using R (version 4.0.3; R Foundation for Statistical Computing, Vienna, Austria).

## Results

The cohort included 349 telehealth visits for 303 patients. For patients who had more than one telehealth visit, only the first one was included in the analysis. The average age of the patients was 59 years, and 182 (60.1%) were female. Among the 303 patients, 50 (16.5%) returned for a face-to-face visit within 30 days of the telehealth visit (95% CI 12.5% to 21.2%) and 71 (23.6%) returned for a telehealth visit within 60 days (95% CI 18.9% to 28.8%) (Table 1).

**Table 1:** Demographics and data for patients returning for care.

Average age (years)	Females	Males	Returned in-office within 30 days for the same complaint	Telehealth visits for different complaints within 60 days	Provider directed in-office visit
59 (19-89)	182 (60.1%)	121 (39.9%)	50 (16.5%)	71 (23.6%)	13 (3.7%)

Cough, anxiety, sinusitis, diarrhea, back pain, and sore throat were the most common diagnoses identified. Among the 32 patients with cough listed in the diagnosis, five (15.6%) returned for a visit within 30 days. Among the 22 patients with anxiety listed in the diagnosis, one (4.5%) returned for a visit within 30 days. Among the 16 patients with sinusitis, one (6.3%) returned for a visit within 30 days. Among the 16 patients with back pain, two (12.5%) returned for a visit within 30 days. Among the 10 patients with diarrhea, 0 (0%) returned for a visit within 30 days. Among the 10 patients with a sore throat, two (20%) returned for a visit within 30 days (Table 2).

**Table 2:** Most common diagnoses seen for a telehealth visit and the number of patients that required a face-to-face visit within 30 days of the telehealth visit.

Diagnosis made during a telehealth visit	Number (N) of patients with the diagnosis	Number (N) that required a face-to-face visit (not provider directed) and diagnosis change (N(%))
Cough	32	5 (15.6%)
Anxiety	22	1 (4.5%)
Sinusitis	16	1 (6.3%)
Back pain	16	2 (12.5%)
Sore throat	10	2 (20.0%)
Diarrhea	10	0 (0.0%)

In the cohort, 16.5% (95% CI 12.5% to 21.2%) returned for a face-to-face visit within 30 days of their telehealth visit. These results suggest that acute care needs can be addressed through telehealth visits and are unlikely to require follow-up face-to-face care. Furthermore, 19 of the 50 patients (38%) that returned for a face-to-face visit did not have a change in diagnosis and, in some instances, the diagnosis made on the telehealth visit was only slightly different from the face-to-face visit (Table 3).

**Table 3:** Diagnosis of patients seen on video visit that required an in-office visit, and the diagnosis made during the in-office visit. GERD: Gastroesophageal reflux disease; ED: emergency department; TMJ: temporomandibular joint

	Diagnosis on telehealth visit	Diagnosis in office	Additional comments
Provider directed			
	Lightheadedness/syncope	Lightheadedness/syncope	
	Abdominal pain/hematochezia	Colitis	
	Shortness of breath	Asthma	
	Fatigue	Fatigue	
	Depression	Depression	
	Gastritis	GERD	
	Abscess	Abscess	
	Rib pain	Rib fracture	
	Abnormal uterine bleeding	Abnormal uterine bleeding	
	Breast pain	Chest pain	
	Cough, COVID-19	Pneumonia, acute hypoxia	This patient was sent to the ED and was hospitalized.
	Cough	Pneumonia	
	Tinnitus	Bilateral cerumen impaction	
Patient initiated			
	Sore throat	Throat pain	
	Swelling hand	Arthritis hand	
	Acute otitis media right	Acute otitis externa right	
	Acute on chronic CHF	Pneumonia	
	Pain thoracic spine	Pain thoracic spine	
	Pain low back	Pain low back	
	Pain low back	Osteoporosis with fracture T9-10 wedge compression sequela	
	Dyspnea	Dyspnea	
	Tinea pedis	Cellulitis	
	Pain low back	Pain low back	
	Sore throat	Rhinitis	
	Anxiety	Anxiety	
	Rhinitis	Rhinitis	
	Numbness hand	Cervical radiculopathy	
	Cough	Mild intermittent asthma	
	Bronchitis	Shortness of breath	Received a prescription for antibiotics on the telehealth visit. The patient ultimately went to the ED.
	Otitis media right/acute pharyngitis	Eustachian tube dysfunction	Received a prescription for antibiotics on the telehealth visit.
	Blood in stool	Hemorrhoids	The patient was sent for a colonoscopy, and the diagnosis was confirmed.
	Acute sinusitis	Bronchitis	Received a prescription for antibiotics on the telehealth visit.
	Left upper quadrant pain	Abdominal wall strain	Received a prescription for omeprazole during the telehealth visit.
	Left ankle pain	Stress fracture left distal fibula	Face-to-face visits led to an MRI confirming the diagnosis.
	Intertrigo	Intertrigo	
	Left thumb pain	Left thumb pain	
	Right otitis media	Right TMJ disorder	Received a prescription for antibiotics on the telehealth visit.
	Cough	Postinfectious cough	
	Cough	Allergic rhinitis	
	Carpal tunnel syndrome	Carpal tunnel syndrome	
	Acute otitis media bilateral	Eustachian tube dysfunction	Received a prescription for antibiotics on the telehealth visit.
	Cough	Dyspepsia	
	Acute sinusitis	Acute sinusitis	
	Generalized anxiety disorder	Acute stress reaction	
	Hematuria	Nephrolithiasis	The patient underwent a diagnostic workup to confirm the diagnosis following a face-to face visit.
	Asthma with acute exacerbation	Cough	
	New headache	Sinusitis	The diagnosis was made on a CT scan that was ordered at the face-to-face visit.
	Herpes simplex labialis	Lichen planus	After a face-to-face visit, the patient was referred to dermatology where the diagnosis was confirmed.
	Edema	Peripheral vascular disease with multiple comorbidities	
	Cervical lymphadenopathy	Cervical lymphadenopathy	
	Strep pharyngitis	Sore throat	Received a prescription for antibiotics on the telehealth visit.
	Bilateral ear pain	Sinusitis	
	Cough	Bronchitis	
	Low back pain	Sciatica	
	Vaginitis	Pruritis vagina	
	Asthma exacerbation	Pneumonia	
	Left knee pain	Left knee pain	
	Gastritis	GERD	
	Abdominal pain/diarrhea	Abdominal pain/diarrhea	
	Neck pain and swelling	Thyroiditis	The patient underwent diagnostic evaluation including labs and imaging to confirm the diagnosis. The workup was suggested on a telehealth visit but started when the patient had a face-to-face visit.
	Low back pain	Muscle spasm	
	Left lower quadrant pain	Left lower quadrant pain	
	Rash hand	Dermatitis	

Of the 71 (23.6%) patients who returned for a telehealth visit within 60 days but greater than 30 days of the initial acute complaint, none were seen for the same complaint.

## Discussion

The utilization of telehealth increased rapidly during the 2020 pandemic and remained a key component to accessing healthcare during a time when many primary care practices were restricting traditional in-office visits. The expansion of telehealth services revealed many of the other benefits of telehealth beyond infection control [13]. Telehealth helped to remove barriers to care including distance, travel restrictions, and the ability to receive care without having to leave or take time off for work. Furthermore, it helped expand primary care services without adding any additional workspace and potentially freeing up exam and office space while physicians worked remotely.

Although research has been done to evaluate the effectiveness of telehealth services on the management of chronic healthcare conditions, little research has been done on how often a face-to-face visit is required following a telehealth visit for acute care issues. Additionally, more information is needed on how effective telehealth visits are for diagnosing and treating acute illnesses and conditions in a primary care practice. The primary aim of this descriptive retrospective study was to evaluate the ability of primary care providers to accurately address acute care needs using a telehealth visit without the need for follow-up in-person care. The results of this research indicate that telehealth appointments can effectively diagnose and treat patients with acute illnesses. Furthermore, in-person follow-up care is often unnecessary. The combination of a large sample size and diverse diagnoses contributes to the significance of this paper. Assessing physicians’, residents’, and advanced practice providers’ (APPs’) use of telehealth services for acute care needs significantly enhances the value of this research.

An incorrect diagnosis in primary care can have a significant impact on the patient, provider, and healthcare system [14,15]. In a previous retrospective study evaluating the diagnostic accuracy of primary care physicians and APPs, in a traditional clinic setting, it was discovered that 21% of the 286 patients seen had received the incorrect diagnosis [16]. The data findings from this research showed that 16.5% (50) of the 303 patients that underwent a telehealth visit returned for an in-office visit within 30 days. Of the 50 patients that returned for an in-office visit, 31 (10.2% of the 303 patients that had a telehealth visit) had a change in diagnosis, and 19 (6.3% of the 303 patients that had a telehealth visit) had an unresolved complaint, but the diagnosis did not change. These findings suggest that telehealth visits can be an effective way of diagnosing and treating patients for acute illnesses and are unlikely to require in-person follow-up care.

Similar findings were found in a study evaluating the differences in diagnosis and treatment using telehealth versus in-person evaluation for pediatric acute illnesses. The patients were either evaluated by a physician in person or via telemedicine, based on random assignment. The primary measure of reproducibility was the agreement on diagnosis between the in-person physician and the telehealth physician. The two groups agreed on the diagnosis for 89% of the 492 visits studied [17].

In a more specific study, orthopedic surgeons compared the diagnostic effectiveness of a telehealth shoulder examination against a traditional shoulder clinical examination for rotator cuff tears using magnetic resonance imaging (MRI) as a reference standard. Of the 62 patients that were evaluated, there was no significant difference between the overall diagnostic effectiveness of the two groups [18].

The most common diagnoses made during the telehealth visits were cough, anxiety, sinusitis, diarrhea, back pain, and sore throat. Except for diarrhea, these align with the most common acute diagnoses seen by primary care physicians in developed countries [19]. Patients with a complaint and diagnosis of cough were the most likely to require an in-clinic visit (5/32; 15.6%) within 30 days of a telehealth visit. This is most likely because of the inability of the provider to perform a physical examination and auscultate the lungs. Conversely, the complaint and diagnosis of diarrhea did not require any in-clinic visits following the telehealth assessment (0/10; 0%). This may be because most infectious causes of diarrhea are self-limiting and do not require a diagnostic evaluation [20].

Of the initial 349 charts that were reviewed, 13 (3.7%) of the patients were directed by the provider to come into the clinic for further assessment. Patients presenting with an ear complaint were the most common reason (3/13; 23.1%) for provider-directed clinic visits. In these situations, the provider needed to perform a physical examination of the ear to accurately make a diagnosis. Identifying the most common reasons patients seek telehealth, the accuracy of diagnosis and treatment, and what complaints require a physical exam allows primary care clinics to develop appropriate scheduling guidelines and workflows for delivering acute care through telehealth visits.

Additionally, 71 (23.6%) patients returned for telehealth visits after their initial acute complaint for other healthcare needs. This suggests that the patients’ experience and confidence with their telehealth visit were favorable enough to utilize the service again within 60 days of their previous visit.

There were some limitations identified that could have an impact on the study results. Initially, the authors solely examined records of patients treated within a specific institution. Consequently, in cases where a patient's symptoms persisted or deteriorated after an initial telehealth consultation, they may have sought medical attention elsewhere. These data would not have been collected during the chart reviews.

Furthermore, there was no differentiation between the type of provider that saw the patient for the telehealth visit. The charts that were reviewed included visits conducted by a staff physician, resident physician, and APPs. The difference in skillset could have impacted the final diagnosis or treatment plan and ultimately whether the patient needed an in-clinic visit. Furthermore, the years of experience were not accounted for during the data gathering. Providers who had longer years of practice may have had better clinical skills and knowledge to assist them in providing adequate diagnosis and treatment. Finally, telehealth experience was not assessed. It is possible that some providers had previous experience with telehealth visits and may have been more comfortable with this model of care.

Additionally, the patients that were assessed during this study did not undergo triage by a clinical staff member before scheduling a telehealth visit and may have been more inclined to undergo a telehealth visit because of the COVID-19 pandemic. By utilizing a clinical triage, the accuracy of telehealth visits may have improved by directing certain complaints to an in-office visit. As healthcare providers become more accustomed to telehealth visits, it will become easier to determine what is appropriate for telehealth services and what issues are better addressed in a traditional clinic setting.

Although this research provides a good assessment of the accuracy of diagnosing acute complaints through telehealth, further research is needed. Having a direct comparison of the diagnostic accuracy of in-office visits with telehealth visits for specific complaints should be assessed to determine if any limitations exist with the use of telehealth.

Additionally, handheld telehealth devices have been developed to allow healthcare providers to connect with patients remotely, in real-time or offline, to enable examination of the heart, lungs, skin, ears, and throat [21]. Further research should be done to evaluate whether the addition of these tools improves the accuracy of diagnosing acute complaints during a telehealth visit.

## Conclusions

The utilization of telehealth, specifically the two-way, synchronous, interactive patient-provider communication through audio and video equipment, appears to be an effective way of diagnosing and treating acute care needs without the need for in-person follow-up care. Telehealth has been shown to increase access to primary care, improve patients’ quality of life, reduce costs, and assist with space constraints. Improvements in data security, patient privacy, virtual waiting rooms, and ease of scheduling have positioned telehealth to remain a vital part of healthcare delivery. As a result, it is recommended that telehealth be utilized by primary care practices to manage chronic conditions and to address acute care needs. However, further research should be done to optimize scheduling guidelines, improve workflows, and to assess the various tools and resources available to improve the accuracy of diagnosing and treating acute care needs.
